# Supplementary material for: Chemical Constituents, In Vitro Antioxidant Activity and In Silico Study on NADPH Oxidase of Allium sativum L. (Garlic) Essential Oil
Source: Antioxidants (Basel). 2021 Nov 20;10(11):1844. doi: 10.3390/antiox10111844 (PMC8615010; doi:10.3390/antiox10111844)
Supplement: Supplementary file 1 [file antioxidants-10-01844-s001.zip › antioxidants-1462227.pdf]

Supplementary material

Antioxidant activity, *In Silico* Study on NADPH Oxidase, and ADMET Prediction of the Chemical Constituents of *Allium sativum* L. (Garlic) Essential Oil

Oscar Herrera-Calderón [1,*], Luz Chacaltana-Ramos [2], Irma Carmen Huayanca-Gutiérrez [2], Majed A. Algarni [3], Mohammed Alqarni [4] and Gaber El-Saber Batiha [5]

1 Department of Pharmacology, Bromatology and Toxicology, Faculty of Pharmacy and Biochemistry, Universidad Nacional Mayor de San Marcos, Jr Puno 1002, Lima 15001, Peru.
2 Department of Pharmaceutical Chemical; Faculty of Pharmacy and Biochemistry, Universidad Nacional San Luis Gonzaga, Av. Los Maestros s/n, Ica 11001, Peru; luz.chacaltana@unica.edu.pe
3 Department of Clinical Pharmacy, College of Pharmacy, Taif University, P.O. Box 11099, Taif 21944, Saudi Arabia; m.alqarni@tu.edu.sa
4 Department of Pharmaceutical Chemistry, College of Pharmacy, Taif University, P.O. Box 11099, Taif 21944, Saudi Arabia; m.aalqarni@tu.edu.sa
5 Department of Pharmacology and Therapeutics, Faculty of Veterinary Medicine, Damanhour University, Damanhour 22515, Al Beheira; Egypt; dr_gaber_batiha@vetmed.dmu.edu.eg

* Correspondence: oherreraca@unmsm.edu.pe; Tel.: (+51-956-550-510)

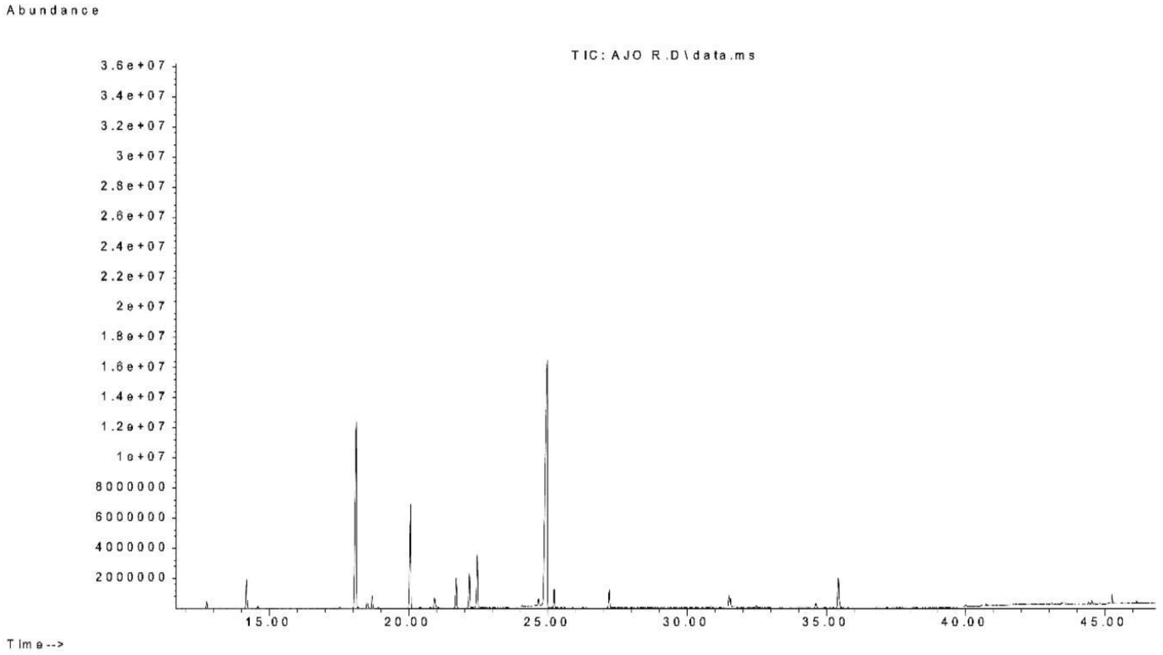

Figure S1. GC chromatogram of the essential oil from garlic

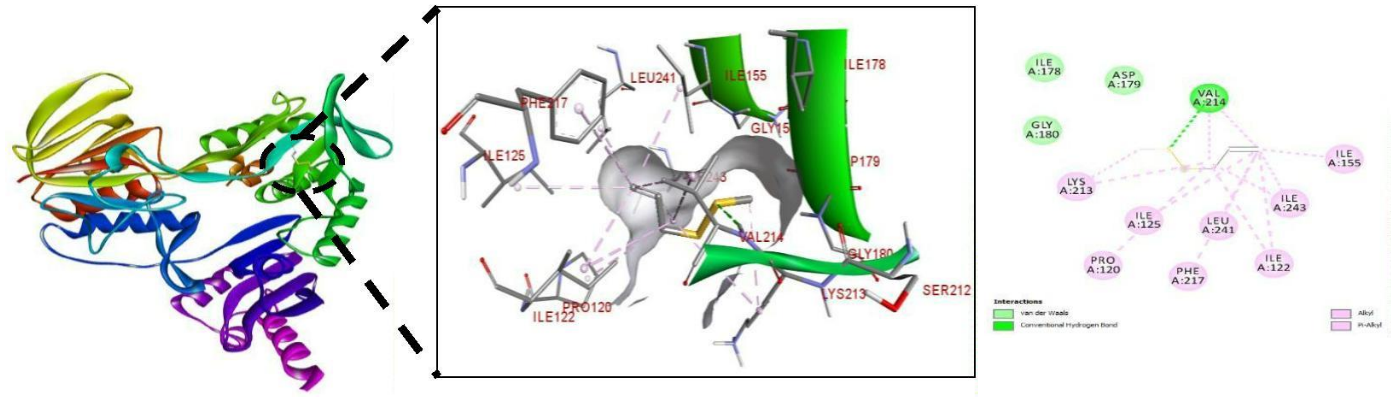

Figure S2. Molecular interaction studies of Allyl methyl disulfide with NADPH-oxidase (PDB ID: 2CDU), surface view (Left panel), and 2D (Right panel) interactions.

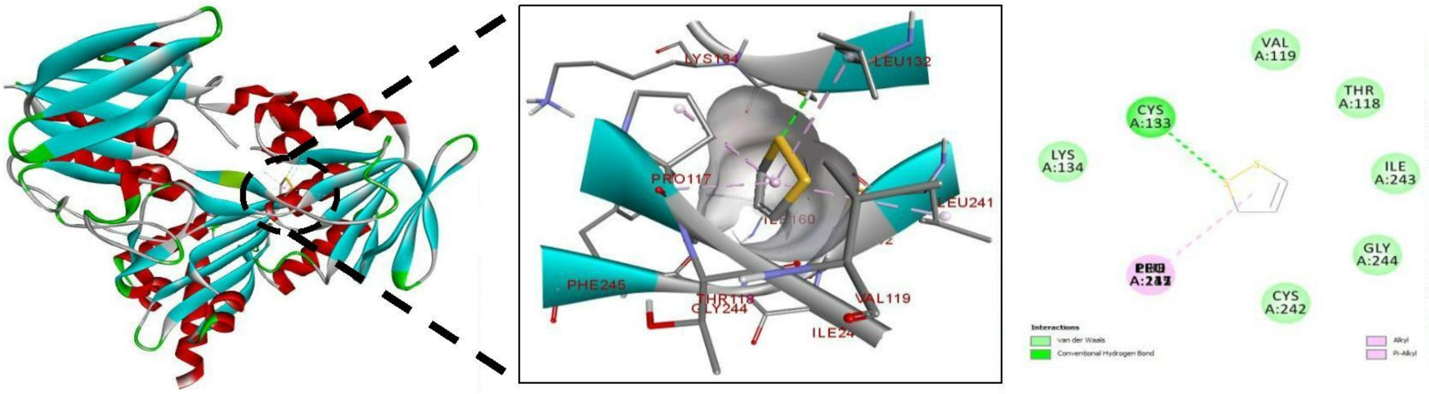

Figure S3. Molecular interaction studies of 3H-1,2-dithiole with NADPH-oxidase (PDB ID: 2CDU), surface view (Left panel), and 2D (Right panel) interactions.

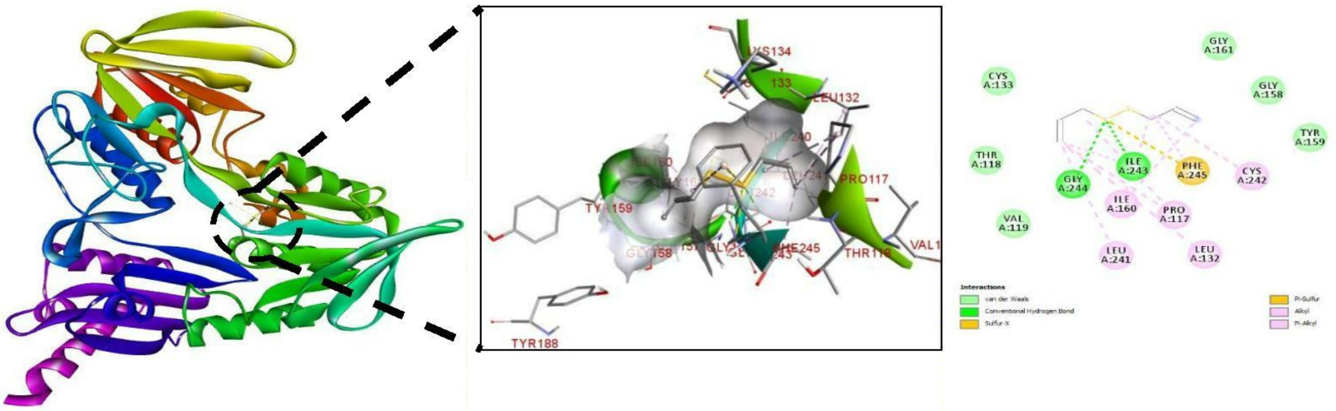

Figure S4. Molecular interaction studies of Diallyl disulfide with NADPH-oxidase (PDB ID: 2CDU), surface view (Left panel), and 2D (Right panel) interactions.

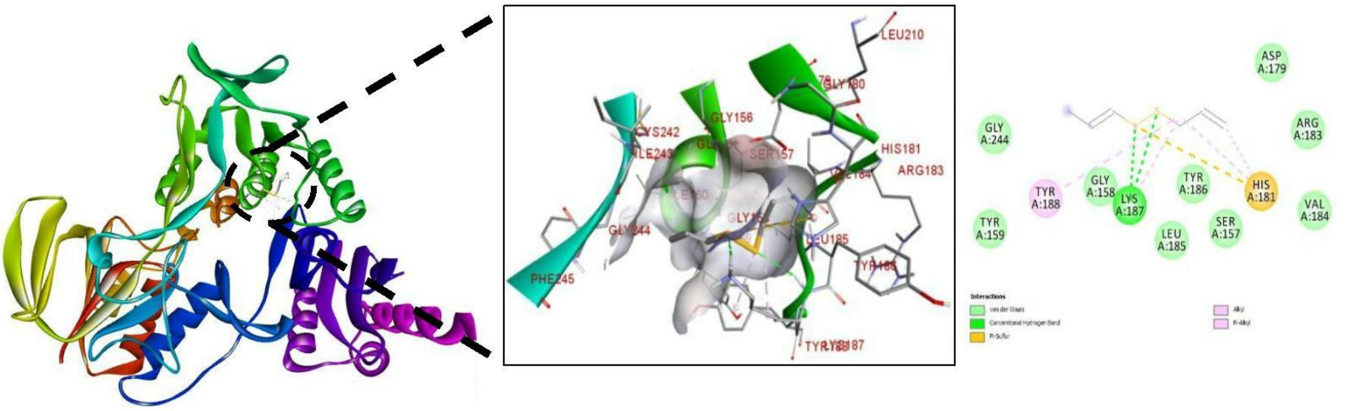

Figure S5. Molecular interaction studies of 1-propenyl 2-propenyl- (E) -disulfide with NADPH-oxidase (PDB ID: 2CDU), surface view (Left panel), and 2D (Right panel) interactions.

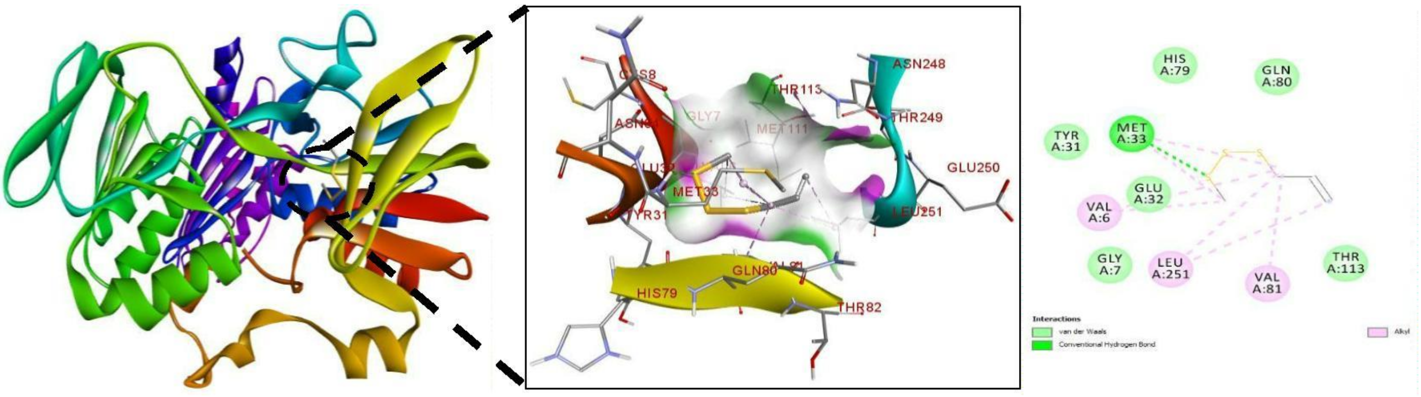

Figure S6. Molecular interaction studies of Allyl methyl trisulfide with NADPH-oxidase (PDB ID: 2CDU), surface view (Left panel), and 2D (Right panel) interactions.

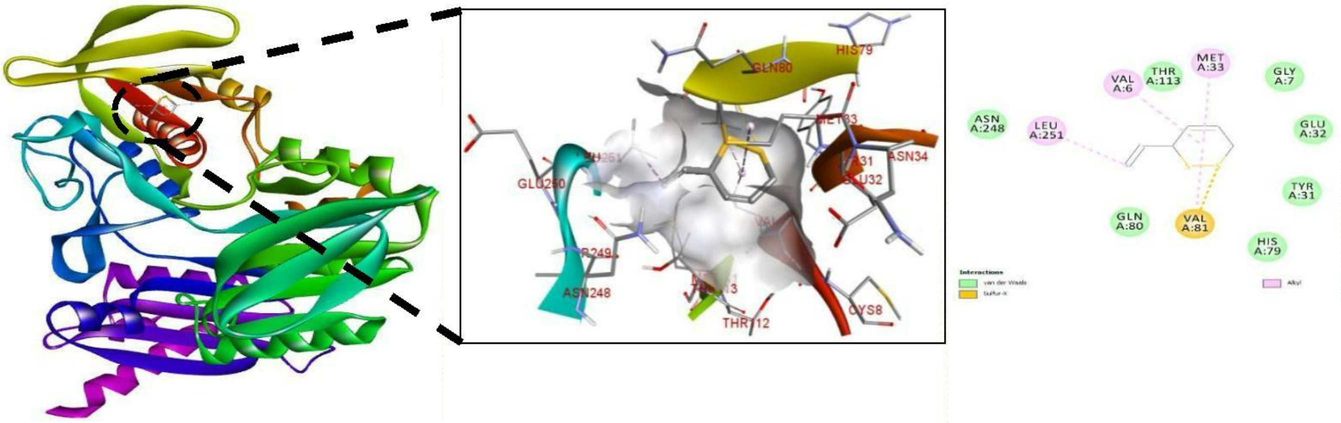

Figure S7. Molecular interaction studies of 3-Vinyl-1,2-dithiacyclohex-4-ene with NADPH-oxidase (PDB ID: 2CDU), surface view (Left panel), and 2D (Right panel) interactions.

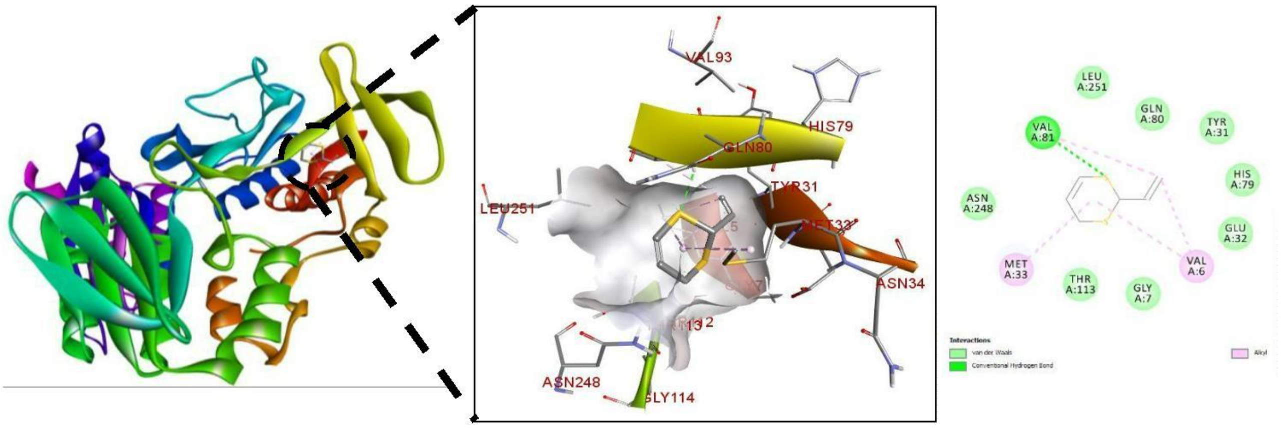

Figure S8. Molecular interaction studies of 2-Vinyl-4H-1,3-dithiine with NADPH-oxidase (PDB ID: 2CDU), surface view (Left panel), and 2D (Right panel) interactions.

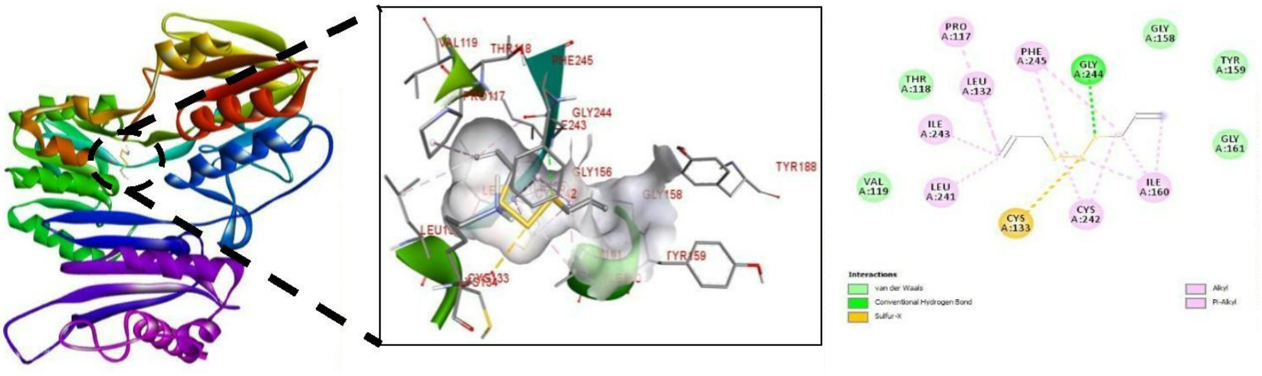

Figure S9. Molecular interaction studies of Diallyl trisulfide with NADPH-oxidase (PDB ID: 2CDU), surface view (Left panel), and 2D (Right panel) interactions.

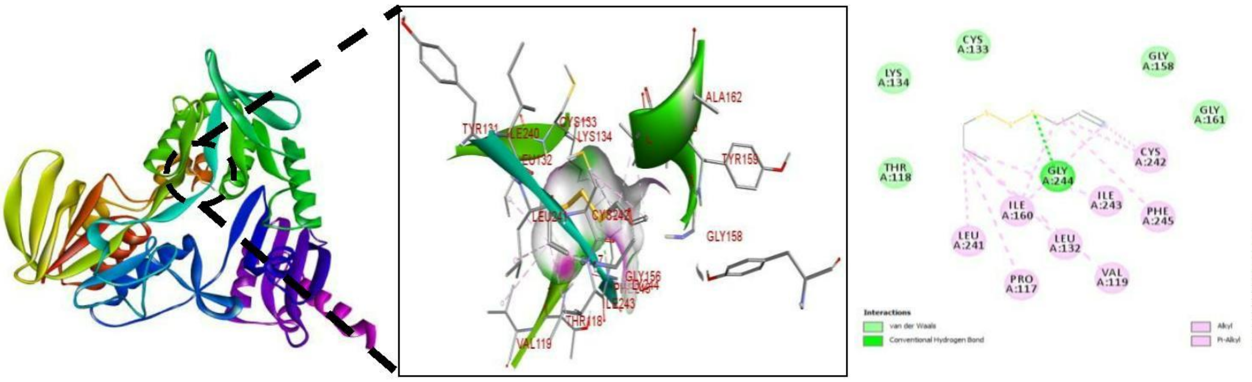

Figure S10. Molecular interaction studies of 1-Allyl-3-propyltrisulfan with NADPH-oxidase (PDB ID: 2CDU), surface view (Left panel), and 2D (Right panel) interactions.

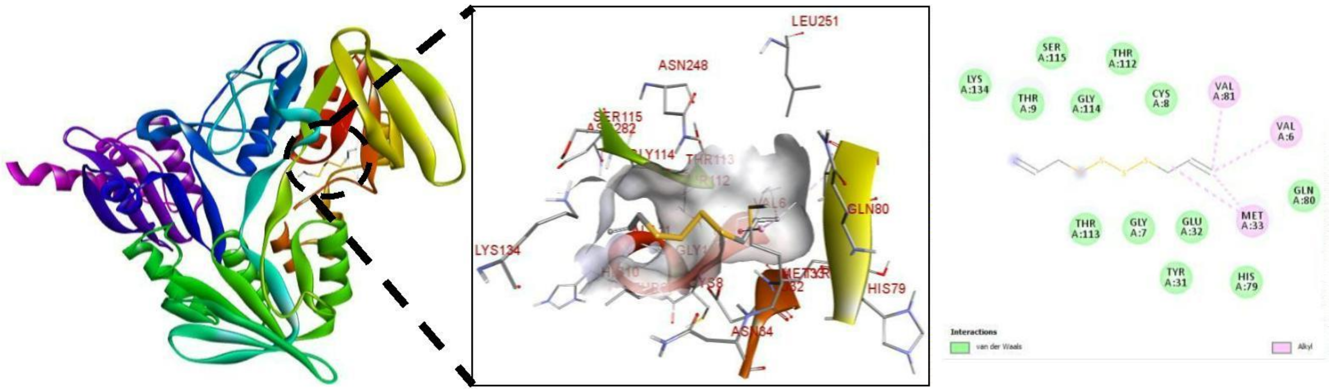

Figure S11. Molecular interaction studies of Diallyl tetrasulfide with NADPH-oxidase (PDB ID: 2CDU), surface view (Left panel), and 2D (Right panel) interactions.
